# Use of Common Salt in Intermittent Fever

**Published:** 1854-05

**Authors:** Joseph C. Hutchison

**Affiliations:** Brooklyn, N. Y.


### ECLECTIC DEPARTMENT.

### *Use of Common Salt in the Treatment of Intermittent Fever.*

BY JOSEPH C. HUTCHISON, M. D., BROOKLYN, N. Y.

The extensive demand for sulphate of quinia, and its consequent high price, and, indeed, the frequent impossibility of obtaining it, in the portion of Missouri where I have recently practised, induced me to subject to the test of experiment various remedies which have from time to time been proposed, as substitutes for that invaluable medicinal agent, in the treatment of intermittent fever.

Among others, a succedaneum, quite recently presented to the notice of the profession, by Drs. Scelle Montdezert and Piorry (viz., common salt,) has claimed our attention, and forms the subject of the present paper.

Although the credit of introducing chloride of sodium, as a remedy for intermittent fever, to the notice of the profession generally, is undoubtedly due to the French physicians above mentioned, it appears that it was used for this purpose prior to that time. Soon after I had commenced experimenting with it, I mentioned the subject to a neighboring physician (a gentleman of undoubted veracity and professional skill), who informed me. that some years previously he had heard of its being used as a domestic remedy for fever and ague by the people in that neighborhood. I also learned, from a highly respectable lady, that her husband's grandfather, many years before, was in the habit of curing fever and ague among his negroes, by giving copious draughts of salt water from a large salt spring on his farm.

Notwithstanding the favorable reports which have been made, the remedy has been but little used in this country, because its antiperiodic influence was doubted. And it has been thought not inappropriate again to introduce the subject to the notice of the profession, with the hope that it may receive more general attention. The experiments were commenced for the purpose of determining, with some degree of accuracy, a knowledge of its value in intermittent fever, by exhibiting it in a large number of cases. And no better field could have been selected for the experiment, not even the far-famed Pontine marshes, whether we consider the very general prevalence of the disease, or the protean and obstinate forms it here sometimes assumes. In consequence, however, of my removal to this city, it was presribed in but twenty-two cases, occurring in twenty individals, a summary of which is now presented as they were recorded; there has, consequently, been no attempt at a selection of the cases. I was enabled to ascertain the permanency of the cures in but three patients, and the condition of the spleen in but one. It would have been very desirable to have observed its effects on the spleen, which, according to

Piorry, is diminished in volume with marvellous celerity. These unavoidable imperfections in the history of the cases arise from the fact, that most of the patients resided at a considerable distance from my office, and were prescribed for without having been examined; and, indeed, from the very general prevalence of the disease, most persons have become so familiar with the use of quinia (which is usually kept in the house, and prescribed as a matter of economy and convenience by the head of the family, when necessary), that a resort to the physician in ordinary cases is rarely thought of. But I think we may safely surmise, that hypertrophy of the spleen existed in every case, excepting, perhaps, in cases 13 and 16, which were recent cases, occurring in very young subjects; because, in malarious districts, this pathological condition exists almost universally, and can be readily detected by careful percussion, even in persons who have never had what are commonly termed miasmatic diseases (intermittent and remittent bilious fevers), and who enjoy apparent good health. It appears to be the direct effect of the malarial poison, and it is a fact well known to practitioners in miasmatic districts, that in all diseases occurring there, even in those not strictly of malarial origin, the morbid effects of this poison are more or less obvious, requiring for their treatment specific remedies, in addition to their ordinary therapeia.

[Twenty-two cases are then detailed. We shall omit these, however, as the author's comments upon them fully exhibit the type, duration and permanency of the cure of the disease; the dose required as well as the mode of administration, &c.]—*Med. Examiner.*

Recapitulation.—*Age.*—9 were under ten years of age, 6 between twelve and twenty, 4 between twenty and forty, and 1 at forty.

*Sex.*—7 were males, 12 females, and 1, sex not known.

*Race.*—16 were white, and 4 black.

*Proportion of Cases cured, benefited, &c.*—Of the 22 cases reported, in 12, or 54.5 per cent., viz., Nos. 1, 3, 6, 7, 8, 12, 14, 16, 17, 19, 20, 21, the paroxysms were immediately suspended. Nos. 12, 20, 21, occurred in the same patient.

In 3 of the cases, or 13.6 per cent., viz., 5, 9, 18, one paroxysm only occurred after the remedy was commenced. It was completely successful, therefore, in 15 cases, 68.2 per cent. In cases 2, 11, 22, the paroxysms were postponed or moderated. No. 11. it will be remembered, vomited after each dose, so that the salt was not retained in sufficient quantity to have produced any marked antiperiodic effect. For Nos. 2, 4, 13, and 15, the remedy was not prescribed a second time, the patients objecting; an increased dose might have arrested the disease. Case 4 did not take all that was prescribed. In one case only (No. 10,) after fair trial, was there no obvious good effect from the remedy.

*Permanency of the Cures.*—In three of the patients only, for reasons which have been elsewhere stated, was I enabled to ascertain with any degree of accuracy the permanency of the cures. Cases 12, 20, 21, which occurred in the same patient, had longer intervals of immunity from the disease each time when checked by the salt, than when quinia had effected the same purpose; and when last heard from, five months had elapsed without a return of the malady. It was said of No. 3, that the disorder did not return so soon as it had previously done when checked by quinia; and of No. 6, it will be remembered, that the patient had not relapsed twelve months after the paroxysms had been checked by nine drachms of the salt, although they had previously returned quite frequently after the use of quinia. So far as the evidence goes, therefore, (which, however, is too limited for a general conclusion,) it indicates the superiority of the chloride of sodium over the usual remedies in the permanency of the cures effected by it. And here we should not lose sight of the favorable influence that may have been exerted by the quinia before the salt was prescribed.

The difficulty of effecting positive cures of intermittent fever by any remedy or course of treatment, however rigidly pursued, is very great, and sometimes impossible, even though prophylactics be continually used, as long as the individual remains exposed to the cause which developed it. The writer can here speak emphatically, because he has, on two different occasions, been compelled to "fly his country" in order to get rid of this harrassing pest. In a number of cases, and among others now distinctly remembered are No. 6 and 7 detailed above, the paroxysms *would* recur every two or three weeks, notwithstanding quinia with Vallet's mass and other remedies to relieve the disordered viscera, including counter-irritation over them, were diligently plied.

*Duration of the Disease, and general Health of the Patients.*—In a large proportion of the patients the disease had existed a very long time. Of most of them it is noted, that they had been its victims from six to twelve months. By this it is not to be understood that the disorder then commenced *de novo*, but that it had recurred more regularly and with shorter intervals during that period than previously; for many of them had been victims for a much longer time, and indeed a few could scarcely remember any period of their lives when they were not from time to time subject to the disease. In four cases (11, 13, 16, 17,) the patients had never had the disorder before; and in most of them (all but the very recent ones,) there was of course more or less impairment of the general health, with visceral obstructions.

*Modus Operandi.*—We have seen that chloride of sodium *does* cure intermittent fever, and now the interesting question arises, What is its *modus operandi?* Upon this subject there is a variety of sentiment. It is the opinion of M. Scelle Montdezert "that paroxysmal fevers arise from the presence of fibrin in the venous blood," and that the salts of quinia, and also *chloride of sodium*, "owe their efficacy as antiperiodics to the fact that they dissolve the fibrin abnormally present, thus restoring the venous blood to its normal conditions." (See Dr. Lattimore's paper.) I suppose he means *excess* of fibrin, or of colorless corpuscles (which, as Mr. Paget remarks, cannot, by any mode of analysis yet invented, be separated from the fibrin of mammalial blood),* or of both, constituting the disease which Prof. Bennet, of

* Kirkes and Paget's Manuel of Philosophy.

Edinburgh, calls, very significantly, *leucocythemia,* and which he has recently described as occurring in cachectic states of the system, attended with organic disease of the lymphatic glandular system, and more particularly of the spleen, which he includes in that class. Not having seen the memoir of Montdezert on this subject, which was presented to the French Academy of Medicine, I am unable to say how his conclusions were arrived at: whether they are the result of a carefully conducted analysis of the blood, before and after using the remedy, or merely a conjecture. It would be necessary, in order that his views be generally adopted, that they should be based on satisfactory evidence. The enlightened state of medical science at the present day, and the rigorous exactness to which it is aspiring, demands that all its facts should rest on the most indubitable evidence of their truth.

It is believed by M. Piorry (who was one of the committee appointed by the Academy to report upon the memoir of M. Scelle Montdezert,) that enlargement of the spleen is the cause of all paroxysmal fevers, and that chloride of sodium cures intermittent fever, like the sulphate of quinia, by acting on the spleen and diminishing its volume, and this sometimes in less than a minute. We are prepared to admit, that the spleen, when of abnormal size, tends to keep up the disease when once contracted, and that it is diminished in volume by sulphate of quinia and chloride of sodium (but not *pari passu*) with the fever; for we feel quite sure that the fever is often cured, whilst the spleen remains moderately enlarged. And the fact which he announces, that "whenever the spleen has a greater length (measuring in a line from the middle of the axilla to the anterior superior spinous process of the ilium) than from 31 to 33 lines, intermittent fever exists," is certainly not true in the malarious districts of this country, if it is otherwise in the field of Piorry's observation. If this were a fact, there are very few of the denizens of our miasmatic regions who would not be doomed victims of the disease; because, as has been already stated, enlargement of the spleen (often to the extent of 31 or 33 lines in its longitudinal diameter) exists almost universally in such localities, in persons who have for some time resided there, even though they may never have had intermittent fever, and *a fortiori* in those who have had the disease, but have for a long time enjoyed an absolute immunity from it.

A theory of its mode of acting, which, it occurs to the writer, is more consistent with reason and established truth, is, that chloride of sodium (being an antidote to the poison which produces intermittent fever, in certain doses) enters the circulation by means of absorption, and neutralizes the miasmatic effluvia which probably operate on the system through this channel; or, in other words, that it developes a condition of the system which is inconsistent with the existence of malarial disease.

We know that bark is an antidote to the miasmatic poison, because, in most instances, its constant use will keep off such diseases from individuals who have been subject to them, and that they return when the remedy is omitted, if the individual continues exposed to the cause which produces them. We can have no better illustration than this of the sequence of cause and effect. Our experience with chloride of sodium in paroxysmal fevers has not yet been sufficient to determine accurately its value, in preventing a recurrence of the attacks when constantly used ; but there is every reason to believe it will prove not much inferior to the preparations of bark, and for the same reason.

Common salt may also act beneficially in intermittent fever by increasing the quantity of red globules in the blood, thereby removing the anemia which is one of the most constant concomitants of the disease. According to the experiments of M. Plouviex, made upon himself at intervals during twenty-five months, a saline regimen has the effect of *increasing the strength and weight of the body*. He began with a teaspoonful daily, which he increased to a tablespoonful, continuing to take this dose on several occasions, for a period of three or four months. The regimen appeared to produce *plethora*. The blood, analyzed while under the full effects of the salt, was found to contain more of the *globules* and salts, but less of the albumen and water.—*United States Dispensatory*.

*Dose and Mode of Administration.*—The quantity given varied from eight to twelve drachms during the apyrexia. At first, eight drachms were given, but the amount was subsequently increased to nine, ten, and even twelve drachms in one instance, with obvious benefit. Children required somewhat larger proportional doses than adults.

Mucilage of elm was selected as the vehicle, on account of its convenience, and because it sufficiently disguised the remedy, which was deemed a matter of importance ; for it would have lost much of its efficacy, or have been repudiated altogether, had the patients known they were taking simply common salt ; as it is well known to physicians that the influence of the mind upon this disease is very considerable. The following was the formula used :

℞ Chloridi sodii ʒiij.

Ulmi pulv. ʒiij.

Aq. bullientis f℥viii.

Infuse two hours and strain. This forms a saturated solution.—Dose, a tablespoonful every two, three, or four hours, so that five or six doses may be taken during the apyrexia. It was not deemed necessary to precede its employment by evacuants, because the patients had recently used such remedies during their former attacks ; and moreover, I preferred to use the salt alone, because its real value could thus be better determined. When it is necessary to precede the use of the salt as an antiperiodic, by emetics or cathartics, perhaps there is nothing better for the purpose, in ordinary cases, than the same remedy administered in emetic doses, which will usually produce also moderate catharsis.

*Disturbing Effects.*—In most of the cases the remedy was well tolerated by the stomach, nausea or vomiting having occurred in but four (3, 11, 14, 15). Four cases also (2, 3, 15, 17,) had moderate alvine evacuations, unattended with pain. There was considerable thirst in every case ; no other unpleasant effects. When given in the above manner (dissolving it in as small a quantity of water as is possible,) it is less likely to disturb the stomach, than the same or even a less amount would in a larger proportion of the solvent. The taste was objected to by some, whilst others disliked it much less than quinia.

*Conclusions.*—From our experience of the antiperiodic virtues of chloride of sodium as detailed above, we think the following conclusions may be legitimately deduced:

I. Although inferior to cinchona and its preparations, it yet forms a *very good substitute* for them in intermittent fever, having failed, as we have elsewhere seen, to produce a speedy suspension of the paroxysms in 31.8 per cent. of the cases only: in a majority of cases therefore it may be substituted for quinia.

II. It may be used instead of, and indeed *preferably* to quinia, first, in cases not unfrequently met with, where the latter remedy is forbidden by the very unpleasant nervous and cerebral symptoms it produced (delirium, tinnitus aurium, cephalalgia, faintness, &c.,) an example of which I have recently seen in the New York Hospital, when sulph. copper was substituted. Secondly, where quinia, from frequent repetition, has lost its effect in ague. Thirdly, it is commended on the *score of economy,* which is a consideration of importance to the poor especially, who are now in a measure debarred from the use of quinia by its high price. And fourthly, it is always at hand, whilst quinia sometimes cannot be obtained.

III. It has been found to be *more energetic* in curing ague than any of the vegetable or mineral tonics commonly used for that purpose, excepting bark, and should therefore be preferred to arsenic, which has been ranked by M. Andral, Prof. Wood, and indeed most other authorities, next in value to quinia. And moreover, I think arsenic should never be used until after quinia and *common salt* have failed to do good, on account of its unpleasant and sometimes disastrous consequences to the general system and stomach, and the increased facilities it affords for using the remedy as a toxicological agent.—*New York Journal of Medicine.*

